# Evolutionary quantitative genetics of behavioral responses to handling in a wild passerine

**DOI:** 10.1002/ece3.945

**Published:** 2014-01-20

**Authors:** Barbara Class, Edward Kluen, Jon E Brommer

**Affiliations:** 1 Department of Biology, University of TurkuTurku, Finland; 2 Museum of Natural History, University of HelsinkiHelsinki, Finland; 3 Aronia Research and Development Institute, Åbo Akademi and Novia University of Applied SciencesEkenäs, 10600, Finland

**Keywords:** Biparental care, Cross-fostering, *Cyanistes caeruleus*, fitness, heritability, personality

## Abstract

Behavioral differences between individuals that are consistent over time characterize animal personality. The existence of such consistency contrasts to the expectation based on classical behavioral theory that facultative behavior maximizes individual fitness. Here, we study two personality traits (aggression and breath rate during handling) in a wild population of blue tits during 2007–2012. Handling aggression and breath rate were moderately heritable (*h*^2^ = 0.35 and 0.20, respectively) and not genetically correlated (*r*_A_ = 0.06) in adult blue tits, which permits them to evolve independently. Reciprocal cross-fostering (2007–2010) showed that offspring reared by more aggressive males have a higher probability to recruit. In addition, offspring reared by pairs mated assortatively for handling aggression had a higher recruitment probability, which is the first evidence that both parents' personalities influence their reproductive success in the wild in a manner independent of their genetic effects. Handling aggression was not subjected to survival selection in either sex, but slow-breathing females had a higher annual probability of survival as revealed by capture–mark–recapture analysis. We find no evidence for temporal fluctuations in selection, and thus conclude that directional selection (via different fitness components) acts on these two heritable personality traits. Our findings show that blue tit personality has predictable fitness consequences, but that facultative adjustment of an individual's personality to match the fitness maximum is likely constrained by the genetic architecture of personality. In the face of directional selection, the presence of heritable variation in personality suggests the existence of a trade-off that we have not identified yet.

## Introduction

Behavioral ecologists consider metrics of behavior as indicative of animal personality whenever behavioral differences between individuals are consistent over time and across contexts (Wilson 1998; Dingemanse and Réale 2005; Réale et al. 2007; Schuett et al. 2010; Stamps and Groothuis 2010a,b2010b). For example, there is between-individual variance (i.e., repeatability) in measures of boldness (Réale et al. 2000), aggression (Bell 2005) or exploration (Dingemanse et al. 2002) in many organisms (reviewed by Bell et al. 2009). Classic theory predicts that facultative adjustment of behavior in different situations maximizes fitness and considers variation among individuals as noise around an adaptive mean (e.g., Krebs and Davies 1978). This view leads to the question why individuals vary consistently in their behavior and how this variation is maintained in a population (e.g., Bell 2007; Wolf et al. 2008). Between-individual variation can be the result of natural selection (cf. Wilson 1998) and phenotypic theoretical models underline that generally framed life-history trade-offs between current and future reproduction could be underlying the emergence of temporally stable behaviors (e.g., Dall et al. 2004; Wolf et al. 2008). Despite the insight provided by such models, these do not specifically identify parameters estimable for empirical workers. For this reason, application of the framework of evolutionary quantitative genetics by behavioral ecologists has been advocated (Dingemanse et al. 2010; Dochtermann and Roff 2010; Brommer 2013a; Dingemanse and Dochtermann 2013). Evolutionary quantitative genetics hinges on understanding two main aspects: (1), the heritability and genetic correlations underlying the (co)variation of traits. (2), selection on these traits. In general, these two aspects provide insight in the response and possible evolutionary constraints acting on natural variation (Falconer and MacKay 1996; Lynch and Walsh 1998).

The evolutionary quantitative genetics framework has thus far shown that the level of individual consistency typically found is largely due to heritable differences in animal personality between individuals. Approximately 30% of variation in animal personality is due to additive genetic effects (Stirling et al. 2002; van Oers et al. 2005; Réale et al. 2007; van Oers and Sinn 2011). Barring substantial interactions between genotype and environmental context in animal personality – an aspect largely unexplored to date (reviewed in Dingemanse et al. 2010; Brommer 2013b) – heritable animal personality implies that individuals are, at least to a certain extent, “hardwired” in their behavior and therefore consistent across time. There is, furthermore, good evidence that the underlying genetic architecture also produces suites of correlated behaviors, so called behavioral syndromes (Sih et al. 2004), by means of genetic correlations (reviewed by Dochtermann 2011). These genetic correlations are typically of such magnitude that they constrain the evolutionary potential of a population to respond to selection and are thus of evolutionary importance (Dochtermann and Dingemanse 2013).

Regarding the second aspect of evolutionary quantitative genetics, we know that many aspects of animal personality are under selection (reviewed in Dingemanse and Réale 2005; Smith and Blumstein 2008). However, the pattern of selection on animal personality is often complex. Meta-analysis shows that bolder individuals have a higher reproductive success, but a shorter lifespan (Smith and Blumstein 2008). It is hence worthwhile to consider both survival and reproductive success as separate selective forces on personality in the same population. Furthermore, the direction of selection on personality may be temporally fluctuating (Dingemanse et al. 2004; Quinn et al. 2009), which is an important element to recognize, as it may facilitate the maintenance of variation in heritable personality (Réale et al. 2010). Lastly, sexual selection is argued to be a considerable force in shaping variation in human and animal personality (Schuett et al. 2010). In particular, in species with bi-parental care, there is evidence that the combination of personalities of both parents has fitness consequences and hence that individuals may gain fitness benefits through selective mate choice. For example, assortative mating in dumpling squid *Euprymna tasmanica* with respect to boldness increased fertilization success (Sinn et al. 2006). In wild great tits *Parus major*, parents that mated assortatively in terms of their exploratory behavior had greater fitness (Both et al. 2005). Cross-fostering in laboratory zebra finches *Taeniopygia guttata* demonstrated that pairs mated assortatively in terms of exploratory behavior had higher fitness because of their greater capacity to foster their offspring compared to disassortatively mated pairs (Schuett et al. 2011). The latter study demonstrates that the fitness benefits of assortative mating need not be genetic, but that the combination of the two parents' personalities can determine the rearing environment (cf. Royle et al. 2010). Taken together, the evidence to date largely suggests that assortative mating improves fitness (reviewed by Schuett et al. 2011), suggesting that (for a variety of reasons) behavioral compatibility of parents is favored (Burley 1983). Nevertheless, under certain conditions, disassortative pairs could be selectively favored (Dingemanse et al. 2004; Dingemanse and Réale 2005; van Oers et al. 2008).

**Figure 1:**
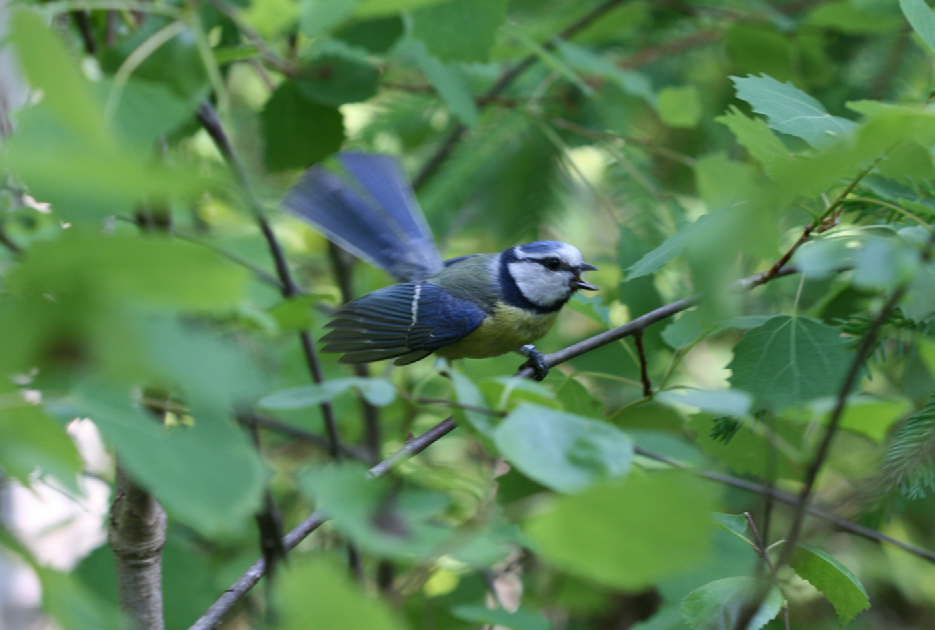
Blue tits are small passerines of approximately 12 g in mass. Blue tits are, in general, aggressive when ranging free and when handling them. This picture shows a male blue tit displaying his bright blue coloration and sounding alarm calls.

In this study, we explore a long-term (2007–2012) individual-based data set on adult blue tit (*Cyanistes caeruleus*; Fig. 1) behavior. Aggression scores and breath rates during handling were measured to quantify these behaviors' heritability and genetic correlation as well as the fecundity and survival selection acting on these. Handling aggression and breath rate are relatively simple field-based metrics of behavior, which can be integrated in the standard measuring protocol, allowing efficient collection of information on each adult individual caught in the population. Offspring may recruit back into the breeding population and, as a consequence, additive genetic (co)variances can be estimated on the basis of resemblance across relatives and fecundity selection pressures can be estimated on the basis of local recruitment. Earlier work has shown that, in adults, handling aggression and breath rate are significantly repeatable (Kluen et al. in press). In blue tit nestlings, handling aggression and breath rate are significantly heritable and negatively genetically correlated (Brommer and Kluen 2012). We, hence, expect these two personality traits to be heritable also in adults and genetically correlated.

Blue tits, like 90% of the other species of birds, provide biparental care to their young (Gross 2005). In such mating systems, fecundity selection on animal personality is typically not only a property of the individual's own personality, but also of that of its partner (Schuett et al. 2010). We therefore explore fecundity selection within a cross-fostering design carried out 2007–2010, where each year, approximately half the nestlings were reciprocally swapped between two broods that were matched in terms of nestling age and size. This design permits us to disentangle genetic and rearing effects of parents' personalities on their offspring's probability of recruitment as well as their interaction. In line with evidence to date, we expect a fitness benefit of assortative mating, possibly largely through rearing effects (Schuett et al. 2010).

Quantification of survival in a wild population requires modeling the probability that an individual has survived but was not captured, which necessitates the use of capture–mark–recapture (CMR) models (Lebreton et al. 1992). In particular, when studying the association between personality and survival, there is a risk that variation in capture probability depends on personality (e.g., bold individuals are caught easier) and thereby confounds survival if not properly modeled (cf. Dingemanse et al. 2004). Based on individual identification of adults in 2007–2012, we use the CMR approach to study whether an individual's survival depends on it personality and whether the direction of survival selection varies over time. We have no specific expectations regarding survival selection on blue tit personality; however, fecundity and survival selection on personality in general are in opposite direction (Smith and Blumstein 2008).

## Material and Methods

### Species, study site, and measures on adults

The blue tit (*Cyanistes caeruleus*) is a small passerine in the family of Paridae, which has a large distribution all over Europe and in parts of Middle East and North Africa. The study was conducted in a population of blue tits breeding in nest boxes set up in an area of approximately 10 km² in a mixed boreal forest area near the city of Tammisaari (60°01 N, 23°31 E). Each year, more than 100 pairs of blue tits breed in these nest boxes. The population was monitored during the breeding season (April–July). Hatching date of a clutch (day 0) was established by daily checking for hatching, starting one day prior to the expected day of hatching, as established following standard methods (detailed in Kluen et al. (2011) for this population). Individuals in this population could be identified through the use of standard metal bird rings attached around one leg with a unique code which was read when handling the individual.

Adults were caught when they were feeding their young, typically when these were around 9 days old. Age was estimated on the basis of the coloration of the greater coverts as either yearlings (hatched last year) or older (Svensson 1992). They were ringed (if unringed), sexed (only females have a brood patch), and morphometric measures were taken. The aggressive response (biting, pecking, flapping its wings) of the bird towards the observer during this period of handling was scored on an interval scale ranging from 1 (completely passive) to 5 (fighting continuously) (Brommer and Kluen 2012; Kluen et al. in press). This handling aggression score thus reflects the propensity of a bird to calm down during the taking of the morphometric measurements. Handling aggression was measured from 2006 onwards. After measures were taken, the bird was held still on its back, and the time it took for the individual to take 30 breaths was measured two consecutive times. Breath rate is calculated by taking the average of these two measurements and expressed as the number of breaths per second (Kluen et al. in press). Rapid breath rate is associated with stress (Carere and van Oers 2004). Breath rate was measured from 2007 onwards.

### Cross fostering and measurements taken of offspring

A reciprocal cross-fostering procedure was carried out between first clutches in 2007–2010, as detailed in Kluen et al. (2011). An equal number of offspring were swapped between two nests (termed “dyad” here) when the offspring in each nest was 2 days old. Blue tits have relatively large broods, mostly 8–13 nestlings, and, thus, multiple offspring were swapped in all cases. The main criteria in deciding which nests form a dyad was that the body mass of the nestlings was approximately equal. If the brood size differed between two nests of a dyad, the number of nestlings swapped between nests was approximately half of the smallest brood size within that dyad. Before being cross fostered, nestlings were individually weighed and marked by clipping a unique combination of their toe nails. Decision on which nestlings were swapped was done random-systematically, by randomly deciding whether the heaviest chick of the nest was cross-fostered or not and then alternating the cross-fostering treatment down the size hierarchy of the brood. Cross-fostering was only carried out between two nests in a dyad, in case the average mass of offspring was similar in both nests. The brood in which a nestling hatched is here termed “nest of origin” and the brood in which a nestling was reared “nest of rearing”. Offspring were ringed when they were 9 days old. When the nestlings were 16 day old, their tarsus was measured using a sliding calliper (to the nearest 0.1 mm) and they were weighed (using a portable digital scale, to the nearest 0.1 g). Nestlings which were caught in subsequent years when breeding as an adult in the study population were considered to have recruited into the breeding population.

### Heritability and genetic correlation

The two personality traits studied here were considered as metric characters which vary in the population. We constructed a linear mixed model which uses information on the relatedness between individuals as derived from the pedigree to estimate the additive genetic variance in addition to other components of variance (animal model; Lynch and Walsh 1998; Kruuk 2004). The bivariate additive genetic (co) variance matrix **G** for handling aggression and breath rate was estimated on the basis of the linear mixed model


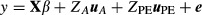
(1)

where **y** is a vector of all observations on all individuals, β is a vector of one or more fixed effects, **X** represents a design matrix (of 0's and 1's) relating the appropriate fixed effects to each individual. The vector ***u***_**A**_ holds additive genetic (random) effects, with *Z*_A_ the design matrix relating the appropriate additive genetic effects to each individual. Similarly, *Z*_PE_ ***u***_PE_ allows for random effect structure on the level of the permanent environment, capturing variance between individuals, which is not due to additive genetic variance, but which is conserved across the repeated records (Lynch and Walsh 1998). For example, permanent environmental variance may be caused by natal or maternal effects and/or variance compounded during the specific environmental conditions encountered by an individual during the time period covered by the measures. In addition, the permanent environmental variance is likely to contain genetic dominance variance as such variance creates “permanent” differences between close relatives (Lynch and Walsh 1998). The additive genetic and permanent environmental effects for handling aggression and breath rate were assumed to be normally distributed with a mean of zero (i.e., defined relative to the trait-specific fixed-effect mean) and with bivariate normal trait-specific variances and one covariance. The matrix **G** (for vector ***u***_**A**_) and its elements (the additive genetic [co]variances) was estimated using information on the coefficient of coancestry *Θ*_*ij*_ between individuals *i* and *j*, as derived from the pedigree. Finally, parameter **e** is a vector of residual errors (difference between observed behavior and the value expected on the basis of fixed and random effects), drawn from a bivariate normal distributions. Hence, the model estimates (co)variances on the additive genetic, permanent environmental, and residual levels. The mixed model was solved using Restricted Maximum Likelihood (REML), as implemented in ASReml-R (Butler et al. 2009; VSN International, Hemel Hempstead, U.K.). The phenotypic variance (*V*_P_) of a trait was calculated as the sum of REML estimates for additive genetic variance (*V*_G_), permanent environmental variance (*V*_PE_) and residual variance (*V*_R_). Heritability (*h*^2^), was expressed by the ratio *V*_A_/*V*_P_ and denotes the proportion of the total variance attributable to the additive effects of genes.

The pruned pedigree (with information on related individuals for which handling aggression and breath rate were measured) holds records of parentage for 562 animals, of which 262 are base parents (phenotyped individuals which have one or more phenotyped descendants but they themselves have unknown parents or whose parents were not phenotyped). Mean family size is 1.6, with 125 full sibs, 46 maternal half-sibs, 49 paternal half-sibs (half-sibs in a social pedigree arise when a parent produces a recruit with a different partner, e.g., in a different year). Lineages of multiple generations are recorded, maximal lineage depth is six generations, but grandparents are identified regularly. This is a social pedigree, where offspring hatched in one nest are assumed to be full-siblings. There are likely to be errors in the paternal links in this pedigree, because some social fathers have not sired the offspring for which they provide care. We do not know the proportion of extrapair paternity in this population, but it is estimated between 7% and 25% in nine populations of blue tits (Brommer et al. 2010). Based on simulation, this level of extra-pair paternity is likely to cause relatively small error in the estimation of the quantitative genetic parameters (Charmantier and Réale 2005). Maternal identity was not known for sufficient individuals to allow modeling variances across mothers.

The fixed effect structure of the model included sex (male or female), age (yearling or older), year of measurement and observer. We included all two-way interactions between sex, age and year. Fixed effects were tested using a conditional Wald *F*-test and random effects were tested using a likelihood-ratio test (LRT), where -2 times the difference in log-likelihood between the model with and without the random effect is tested against a χ² distribution with one degree of freedom.

### Survival analysis

To study survival selection on personality traits, we tested whether the individuals' probability of survival from 1 year to the next was associated to a personality trait. We used the program MARK (White and Burnham 1999) and fitted logistic models to test whether probabilities of survival (Φ) and capture (*p*) of individuals depends on handling aggression or breath rate (analyzed separately), sex, year, and all their interactions. The first measure of each personality trait was considered as the individual covariate. In such analyses, it is crucial that the encounter history of each individual identified in the population is entered, and we, hence, assigned the mean sex-specific personality value as individual covariate for those individuals for which this information was missing (Cooch and White 2012).

We confirmed that our data adhered to the underlying assumptions of the Cormack-Jolly-Seber (CJS) model by performing Goodness of fitting tests (GOF) using the program RELEASE (tests 2 and 3 gave *P*-values of 0.31 and 1, respectively). Tests were based on the most complete model which included effects of sex and years, because MARK cannot take into account individual covariates in GOF tests. We found underdispersion in the data (c-hat = 0.88), but, following standard (conservative) practices, we do not correct survival models for this underdispersion (Cooch and White 2012). In order to test whether different variables (sex, year, personality trait) and their interaction have an effect on the probability of capture and survival of adults, we first run all the possible models for each personality trait separately. Candidate models for each personality trait here included constant, the effect of sex (*s*), year (*t*), personality (*agg* or *br*), and their two- and three-way interactions, which thus defined 19 possible model structures for each personality trait. Inference in CMR modeling were based on an information theoretical approach (Burnham and Anderson 1998) using Akaike information criterion (AIC*c*). As a general rule, a parameter was considered important if adding this parameter leads to a decrease of more than 2 AIC*c*. In step 1, we selected the best model out of the 19 possible candidate models for capture probability *p* (i.e., the model with the lowest AIC*c*), while keeping the full model specifications (i.e., main effects and all interactions) for Φ. We, then, in step 2, selected the best out of 19 candidate models for Φ, while defining *p* as in the best model under step 1. We confirmed that qualitatively the same ranking of candidate models for Φ was observed when *p* was defined according to the second and third best model in step 1 (cf. Karell et al. 2011 for this modelling approach). Although the full set of models was fitted, we, here, tabulate a reduced AIC*c*-based ranking of the candidate model set omitting model variants which have a higher AIC*c* than a hierarchically simpler model version. For example, Φ(*t*) is a hierarchically simpler model than Φ(*t *+ *s*), and whenever the latter has a higher AIC*c* value than the former, it is not an interesting model to report as the inclusion of the extra parameter *s* has not improved model fit as judged by AIC*c*. This reduced set of candidate model avoids redundancy and eases the interpretation of the results (Arnold 2010).

### Recruitment selection estimated on the basis of a reciprocal cross-fostering design

We considered the probability of recruitment of an offspring as an important aspect of an individual's fitness. Our main focus here is to separate the effect of the personality of the genetic parents from the effect of the foster parents on recruitment. We therefore included only information on broods adhering to a fully crossed design, denoting whether a nestling recruited (1) or not (0) in broods that were reciprocally cross-fostered and for which the focal personality trait (handling aggression or breath rate) of both parents was quantified. We modeled the probability of recruitment of offspring using a generalized linear mixed model (e.g., Bolker et al. 2008), assuming binomial errors and a logit link. The model was implemented in the “glmer” function in the “lme4” package (Bates 2005) in R. In order to properly include the structure of the data following from the experimental design, we included nest of origin and nest of rearing as random effects to control for heterogeneity across these levels. Separate analyses were conducted for the two personality traits. The genetic and foster parents' personality trait (handling aggression or breath rate), the interaction between genetic parents' personality and between foster parents' personality, and year were included as fixed effects. The individual's value for the personality was the first measure taken in each year considered. Prior to analysis, each covariate was standardized by its overall mean and standard deviation in order to allow direct comparison of the effect sizes of the fixed effects and to properly model the interaction. Recruitment selection for (dis)assortative mating was modeled by the interaction of the standardized trait values for males and females which formed a pair. Hence, this interaction compares offspring recruitment as a function of parents' personality scores compared to what is expected when both parents have average personality scores. A positive coefficient for this interaction thus indicates that both parents with an above-average personality score enjoy higher fitness than the “average pair” (i.e., selection for assortative mating). The statistical significance of fixed effects was calculated by comparing models with and without each variable using LRT, where the likelihood was approximated using Laplace integration (e.g., Bolker et al. 2008).

### Natal dispersal

One caveat in using local recruitment as an estimate of fecundity in analyzing selection on a behavioral trait is that natal dispersal (which influences the probability to recruit locally in the breeding population) may covary with the focal behavior. For example, natal dispersal distances of great tits covary with exploratory behavior (e.g., Dingemanse et al. 2003, Korsten et al. 2013). For personality traits which were associated with local recruitment, we checked whether offspring of parents of different personalities could differ in their dispersal distances. For each recruit, the distance between the nest box where it was reared and the nest box where it was recorded breeding for the first time was calculated as the Euclidian distance in meters based on the boxes' coordinates (obtained using Global Positioning System). This natal dispersal distance was used as the response variable in a linear mixed model which included their foster and rearing parents' relevant personality traits in order to test whether parental personality scores affected their offspring's dispersal.

## Results

### Heritability and genetic correlations between traits

Each behavioral trait was measured more than 800 times in over 500 individuals in total (Table 1). Handling aggression had more observations than breath rate because it was measured also in 2006. Approximately 61% of individuals were measured only once (Table 1), and these were, hence, not informative in the estimation of permanent environmental (co)variances. Both traits had significant heritability (the random effect “Genetic” in Table 2). The proportion of phenotypic variance due to permanent environment effects was low for handling aggression, but relatively high for breath rate (Table 2). The residual variance explained approximately 60% of the phenotypic variance in both traits (Table 2). The fixed effects revealed clear annual variation, and the quantification of both traits differed between observers (Table 2). In addition, males had a higher handling aggression score and lower breath rate than females. Handling aggression (but not breath rate) was affected by two-way interactions, the most important of which was that yearling females were less aggressive than yearling males.

**Table 1:** Data structure of the two metrics of behavior analyzed. Handling aggression is a score from 1 to 5 describing how aggressive an individual is during handling. Breath rate is the number of breaths per second measured during handling. For each trait, we present the total number of observations (*N*_obs_) and the number of individuals measured (*N*_ind_) with between parentheses the number of individuals with 1, 2, 3, 4, or 5 repeated observations in the data. Per individual, only the first measure of each trait during each breeding season is included. The mean, standard deviation (SD) and range are provided.

Behavior	*N*_obs_	*N*_ind_ (1/2/3/4/5)	Mean	SD	Range
Handling aggression	885	546 (334/129/49/24/10)	3.03	1.11	1–5
Breath rate	822	507 (311/112/59/15/10)	2.26	0.39	1.5–4.1

**Table 2:** Linear mixed model analyses of the two personality traits handling aggression and breath rate. For each trait, all the random and fixed effects included in the mixed model are presented. The estimated variance as well as the proportion of the REML phenotypic variance is given for the residuals and the two random effects, where “Genetic” specifies the additive genetic variance (covariance across relatives) and “Permanent environment” the variance across individuals due to other factors than additive genetic ones. The proportion of REML phenotypic variance due to additive genetic effects gives the trait”s heritability *h*^2^, the statistical significance of which is tested using a Likelihood Ratio Test. Results are here presented for univariate analyses, but are qualitatively the same in the bivariate analysis. Fixed effects were tested with an unconditional *F*-test where the residual degrees of freedom were numerically estimated. Significant fixed effects are indicated in bold. Raw data phenotypic SD is reported in Table 1 and this information can be used to calculate the raw-data heritability. Contrasts are reported whenever relevant and interpretable. The sex-specific contrast is “male”, which specifies the difference in trait value of a male relative to a female, “yearling” is relative to “≥1-year old”, “yearling male” is relative to all the other age/sex classes.

Trait/type	Source	Estimate ± SE	Proportion (SE)	Test	*P*
Aggression	REML phenotypic	1.08 ± 0.059			
	Residual	0.62 ± 0.046	0.57 ± 0.045		
Random	Permanent	0.089 ± 0.072	0.082 ± 0.067		
Random	Genetic	0.37 ± 0.081	0.346 ± 0.066	χ^2^ = 34.8	<0.001
Fixed	**Intercept**	2.49 ± 0.21		*F*_1, 200.4_ = 3371.0	<0.001
Fixed	**Year**			*F*_6, 712.4_ = 4.23	<0.001
Fixed	**Sex** (male)	0.37 ± 0.27		*F*_1, 520.8_ = 51.6	<0.001
Fixed	**Observer**			*F*_5, 751.3_ = 7.92	<0.001
Fixed	Age (yearling)	–0.77 ± 0.25		*F*_1, 762.0_ = 2.65	0.10
Fixed	Sex^*^Year			*F*_6, 716.3_ = 2.37	0.02
Fixed	Sex^*^Age			*F*_1, 771.0_ = 4.19	0.04
	Yearling male	0.85 ± 0.37			
Fixed	Year^*^Age	0.0164 ± 0.0440		*F*_6, 761.6_ = 2.68	0.01
Breath rate	REML phenotypic	4.37 ± 0.241			
	Residual	2.55 ± 0.20	0.58 ± 0.047		
Random	Permanent	0.92 ± 0.35	0.21 ± 0.078		
Random	Genetic	0.90 ± 0.32	0.20 ± 0.071	χ^2^ = 9.3	0.002
Fixed	**Intercept**	2.51 ± 0.022		*F*_1, 145.0_ = 1752	<0.001
Fixed	**Year**			*F*_5, 671.0_ = 5.60	<0.001
Fixed	**Sex** (male)	–0.32 ± 0.03		*F*_1, 469.4_ = 36.9	<0.001
Fixed	**Observer**			*F*_5, 726.1_ = 16.4	<0.001
Fixed	Sex^*^Year			*F*_5, 660.4_ = 1.66	0.1
Fixed	Sex^*^Age			*F*_1, 743.8_ = 3.2	0.07
Fixed	Year^*^Age			*F*_5, 731.5_ = 1.05	0.38

We then constructed a bivariate linear mixed model with the same fixed and random effect structure as in Table 2, but also allowing for covariances between traits. The correlation between handling aggression and breath rate at the phenotypic level as estimated by this bivariate mixed model was approximately zero (0.055 ± 0.039). The genetic correlation also had an estimate close to zero (–0.0227 ± 0.20). The permanent environmental correlation was −0.370 ± 0.46 and the residual correlation 0.175 ± 0.053. Hence, both genetic and permanent environmental correlations had relatively large uncertainty. The residual correlation between these traits was clearly positive (cf. Kluen et al. in press, and discussion therein). Based on these animal model analyses, we conclude that handling aggression and breath rate are heritable aspects of blue tit personality, which are not genetically correlated.

### Survival analysis

**Figure 2:**
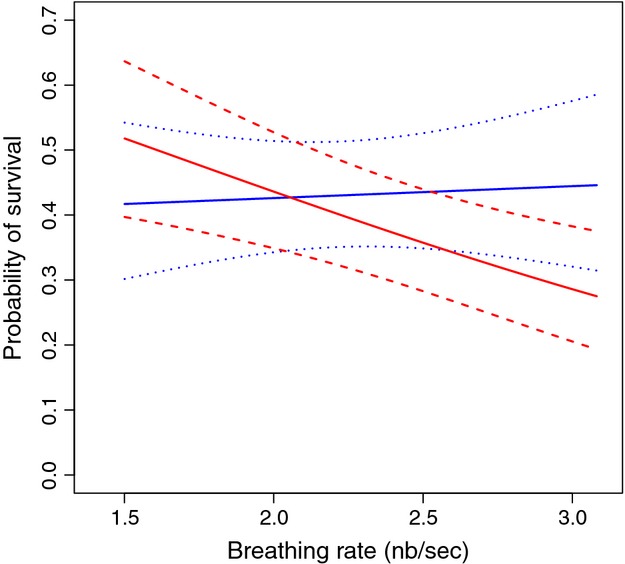
Probability of apparent survival of adult blue tit males and females as a function of their breath rate (*n* of breaths/s) based on capture–mark–recapture (CMR) analysis of encounter data covering 2007–2012, as reported in Table 2. For clarity, we plot the survival selection only for the year 2007, but this pattern was the same in the other years (except for differences in average survival between years), as there was no significant interaction with year (Table 2). Solid lines represent the values estimated by the binomial model for males (blue) and females (red) and the dashed lines represent the 95% confidence intervals. The CMR analyses was based on values of breath rate standardized to zero mean and unit SD, but we, here, plot the relationship of apparent survival and breath rate on the data scale, based on values (1.4–3.0 breaths/s) which contain 95% of its observed distribution.

Capture–mark–recapture analysis of survival was based on the encounter history of 896 adults (409 males, 487 females) for the breeding seasons 2007–2012. For both, handling aggression and breath rate, constant probability of capture had the best model fit [ranking of candidate models in Table S1; capture probability *p* = 0.87, 95% CI: [0.80, 0.91] (Table S2)]. In general, there were clear differences in apparent survival between years (*t* in Table 3), and weak evidence for males having a higher apparent survival (*s* in Table 3; inclusion of sex led to decrease of 0.2 AIC points, see Table S2 for estimates of effect size). We found no evidence of an effect of handling aggression on the probability of apparent survival (Table 3). Models which included handling aggression always had a higher AIC*c* score compared to structurally the same models excluding handling aggression. In contrast, there was a clear effect of breath rate on apparent survival with a complicated pattern, as the top model included an interaction between breath rate and sex on the probability of apparent survival. Inclusion of breath rate and of the interaction between breath rate and sex each led to a decrease of more than 2 AIC*c* (Table 3). Inspection of the model's coefficients (Table S2) revealed that the probability of survival of males did not depend on breath rate, but that females with a fast breath rate had a lower survival (plotted in Fig. 2 for the year 2007; male slope = 0.018 (95% CI: [–0.144, +0.179]), female slope = –0.154 (95% CI: [–0.28, –0.014]).

**Table 3:** Model selection for the adults' probability of apparent survival (Φ) between breeding seasons, as a function of sex (*s*), year (*t*) and personality trait (handling aggression *agg* or breath rate *br*). For all the traits, the probability of capture (*p*) is constant (Tables S1 and S2). Models are sorted by ascending order of AIC*c*. Full candidate model set for Φ included 19 models, but models which have a higher AIC than a hierarchically more simple model are not shown in this summary. Parameter estimates for the top CMR model for breath rate are presented in Table S3.

Φ	*N* of parameters	AIC*c*	ΔAIC*c*
Aggression			
*t *+ *s*	7	1587.7	0.0
*t*	6	1587.9	0.2
Constant	2	1623.9	36.3
*s*	3	1624.2	36.5
*agg*	3	1625.4	37.7
Breath rate			
*t *+ *s *+ *br* + (*s *× *br*)	9	1581.8	0.0
*t *+* br*	7	1583.3	1.5
*t *+ *s* + *br*	8	1584.4	2.6
*t *+ *s*	7	1587.7	5.9
*t*	6	1587.9	6.1
*br*	3	1621.0	39.2
Constant	2	1623.9	42.1
*s*	3	1624.2	42.4

### Recruitment analysis and assortative mating

During 2007–2010, the handling aggression of both parents was recorded in 238 reciprocally cross-fostered broods with a total of 2518 nestlings. In general, the probability of recruitment varied across years (Table 4). For the offspring included in this analysis, recruitment rates were 6.6% (41/623) in 2007, 6.9% (40/578) in 2008, 3.1% (22/710) in 2009, and 4.1% (25/607) in 2010. Note that local recruitment of offspring was recorded up to and including 2012 and hence all offspring produced during the breeding seasons 2007 –2010 were likely to be recorded as breeding adults during the course of this study.

**Table 4:** Effects of the genetic and foster parents' values for handling aggression (HA) on offspring recruitment into the breeding population (*n* = 2518 fledglings from 238 broods). The model (GLMM with a binomial error distribution) includes nest of origin and nest of rearing as random effects. All the individual covariates were standardized to zero mean and unit standard deviation. Year is a 4-level factor. Model likelihood was based on Laplace approximation. *P*(*z*) values are given by a *z*-test on the coefficients to test whether they differ significantly from 0. Coefficients for Year are in comparison with the year 2007. A Likelihood ratio test (χ²) was used to test the significance of the fixed effects by comparing the Laplace approximated likelihood of models with and without each variable.

Effect	Variance	Estimate	SE	*z*	*P*(*z*)	χ²	df	*P*
Random effects								
Genetic ID	0.24					0.97	1	0.32
Rear ID	0.38					2.90	1	0.09
Fixed effects								
**Intercept**		*−***3.04**	**0.23**	*−***13.41**	**<0.001**			
**HA Foster father**		**0.23**	**0.12**	**1.98**	**0.05**			
HA Foster mother		*−*0.10	0.12	*−*0.88	0.38			
HA Genetic father						0.99	1	0.32
HA Genetic mother						0.20	1	0.66
HA Foster father ^*^ HA Foster mother		**0.22**	**0.10**	**2.10**	**0.04**	**5.90**	**1**	**0.02**
HA Genetic father ^*^ HA Genetic mother						0.65	1	0.42
**Year**						**9.79**	**3**	**0.02**
2008		0.02	0.32	0.07	0.95			
**2009**		*−***0.81**	**0.36**	*−***2.29**	**0.02**			
2010		−0.64	0.36	−1.78	0.08			
HA Foster father^*^Year						3.19	3	0.36
HA Foster mother^*^Year						5.34	3	0.15
HA Genetic father^*^Year						2.22	3	0.53
HA Genetic mother^*^Year						5.40	3	0.15
HA Foster father^*^HA Foster mother^*^Year						3.83	3	0.28
HA Genetic father^*^HA Genetic mother^*^Year						1.02	3	0.80

Statistically significant effects and coefficients are indicated in bold.

**Figure 3:**
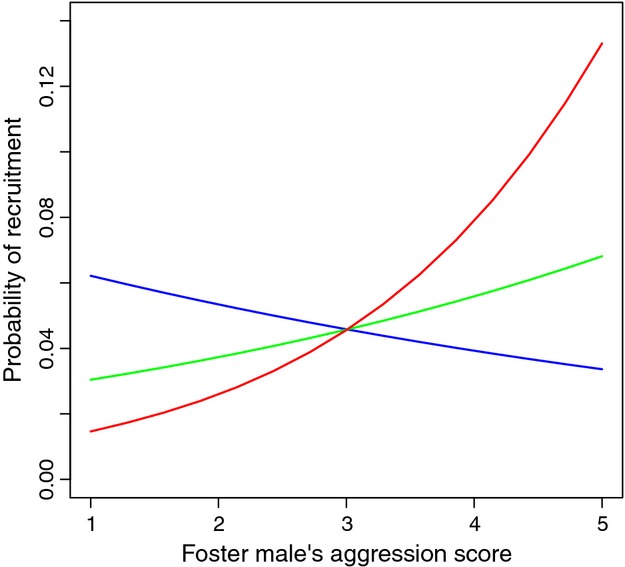
Illustration of the effect of foster parents' handling aggression on the offspring's probability of recruitment as based on reciprocal cross-fostering carried out in 2007–2010, derived from the model coefficients reported in Table 3. Recruitment selection is plotted here for the year 2007 only, but is qualitatively the same in other years since there was no interaction with year (Table 3). The analysis was based on handling aggression standardized to zero mean and unit SD, but is here plotted on the data scale. The probability of recruitment was calculated for foster fathers paired with highly aggressive (score = 5, red), intermediate (score = 3, green) and nonaggressive (score = 1, blue) females.

**Figure 4:**
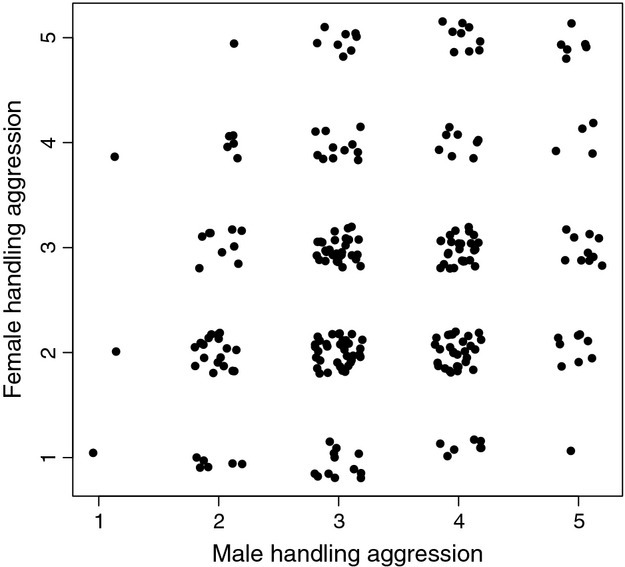
Handling aggression of females plotted against handling aggression of their male partner. Handling aggression is a score of 1–5, and a small random number was added to both axes in order to aid in separating the data points. *N* = 250 pairs. Correlation between the partners' handling aggression (i.e., the degree of assortative mating) is reported in the main text.

The foster (but not genetic) father's handling aggression score increased the offspring's probability of recruitment (Table 4, Fig. 3). In addition, the handling aggression of the female with which the foster male had partnered affected offspring's probability of recruitment in a manner which favored assortative mating (Table 4, Fig. 3). Pairs where both birds had high handling aggression scores were particularly likely to recruit offspring, because of combination of the positive effect of male handling aggression and of assortative mating. Birds mated assortatively according to handling aggression score (Fig. 4, Pearson's *r* = 0.19, 95% CI: [0.067, 0.31], *n* = 250 pairs).

A higher mass at fledging can explain a higher postfledging survival in small passerines. We tested whether the parents' handling aggression had an effect on the offspring's mass at day 16. We found significant differences between years, and a positive effect of the nestling's tarsus length and of the foster father's handling aggression on their offspring's mass at day 16 (Table 5). The relationship found between the parent's personality traits and the probability of recruitment can be confounded with the influence of the parents' personality on the offspring's natal dispersal in case personality and natal dispersal distance are correlated (Dingemanse et al. 2003). Nevertheless, foster parents' handling aggression did not influence offspring natal dispersal distance in the study site (Table 6). We, therefore, conclude that the foster father's handling aggression is associated with improved rearing conditions of his offspring leading to increased fledgling mass, and that the effect of his handling aggression on offspring's recruitment probability is not confounded by an effect on natal dispersal distance.

**Table 5:** Analysis of mass (in grams) of nestlings at day 16 as a function of foster parents' handling aggression (HA). Data consists of the same broods and adults as the recruitment analysis reported in Table 4, although not all nestlings included in that analysis survived to day 16 (*N* = 2098 nestlings, produced by 188 females, 181 males). The identity of foster father and foster mother were included as random effects, explaining 26% and 21% of the total REML variance (0.63). The significance of the fixed effects reported were based on a Likelihood Ratio Test between mixed models (solved using Maximum Likelihood) where the focal variable was excluded compared to the mixed model where it was retained. The interaction was tested first and was removed such that all single terms were tested against a model without the interaction. Contrasts for the factor “year” were given in comparison to 2007. Nestling tarsus length was standardized to zero mean prior to analysis (unit is mm). Handling aggression was standardized to zero mean and unit SD.

Fixed effect	Estimate ± SE	*z*	χ^2^	df	*P*
**Intercept**	10.9 ± 0.070	155.3			
**Tarsus**	0.71 ± 0.027	26.4	601.9	1	<0.001
**Year**			129.8	3	<0.001
2008	0.99 ± 0.085	11.6			
2009	0.89 ± 0.081	11.0			
2010	1.06 ± 0.095	11.2			
**HA Foster father**	0.096 ± 0.033	2.95	8.74	1	0.003
HA Foster mother	–0.048 ± 0.033	–1.44	2.1	1	0.14
HA Foster father^*^ HA Foster mother	–0.020 ± 0.030	–0.68	0.43	1	0.51
HA Foster					
mother					

Significant variables are indicated in bold.

**Table 6:** Analysis of natal dispersal (in m) as a function of foster parents' handling aggression (HA). Data consists of the same broods and adults as the recruitment analysis reported in Table 4, although not all nestlings included in that analysis recruited (*N* = 128 nestlings, produced by 81 foster mothers, 76 foster males). The identity of foster father and foster mother were included as random effects, explaining 0% and 30.2% of the total REML variance (488768). The significance of the fixed effects reported were based on a likelihood ratio test between mixed models (solved using maximum likelihood) where the focal variable was excluded compared to the mixed model where it was retained. The interaction was tested first and was removed such that all single terms were tested against a model without the interaction. Sex gives the contrast of male to female. Handling aggression was standardized to zero mean and unit SD prior to analysis.

Fixed effect	Estimate ± SE	*z*	χ^2^	df	*P*
**Intercept**	1597.4 ± 143.0	11.2			
**Sex (male)**	–486.5 ± 128.9	3.8	15.2	1	<0.001
Year			2.1	3	0.54
HA Foster father	23.4 ± 54.3	2.4	0.1	1	0.72
HA Foster mother	–55.7 ± 62.8	–0.9	0.6	1	0.43
HA Foster father^*^ HA Foster mother	29.5 ± 54.3	0.5	0.35	1	0.55
HA Foster mother					

Significant variables are indicated in bold.

Offspring recruitment was not associated in a systematic fashion to the genetic and foster parents' breath rate (Table S3, Fig. S1).

## Discussion

We find that two field-based metrics of behavior during handling (aggression and breath rate) are heritable and genetically uncorrelated aspects of adult blue tit personality. Both traits also are associated with differential individual performance and are therefore measuring a behavior which is relevant to the individual's performance in the wild. Offspring fostered by a male with a high handling aggression score have a higher probability to recruit than offspring fostered by a male with a low handling aggression score. In addition, offspring fostered by parents that mated assortatively with respect to handling aggression have a higher recruitment probability than those offspring fostered by disassortatively mated parents. These latter results are in good agreement with the notion that parents with a similar personality enjoy fitness benefits (Both et al. 2005; Sinn et al. 2006; Schuett et al. 2011; reviewed by Schuett et al. 2010). Because of our cross-fostering design, we can demonstrate here that the recruitment benefits of male handling aggression and assortative mating stem from the fostering capacity of individuals and are not due to genetic benefits. This has previously been demonstrated in laboratory zebra finches (Schuett et al. 2011), but is – to our knowledge – the first evidence that behavioral compatibility of parents improves rearing capacity also in the wild.

We further find that females (but not males) which breathe faster during handling have a lower annual survival. A fast breath rate during handling is considered a sign of stress (Carere and van Oers 2004; Fucikova et al. 2009; David et al. 2011). We can, at this point, only speculate about why breath rate during handling correlates to female survival. The absence of an effect of breath rate on the probability of capture could indicate that breath rate does not affect predation risk. In winter, blue tits and other tit species jointly form foraging flocks (Dhondt and Eyckerman 1980). Females are competitively inferior to males (Nilsson et al. 2011), and females with a high breath rate are possibly outcompeted during foraging in winter by females with a low breath rate. Such a competitive ranking could explain why breath rate affects female, but not male, survival.

### Genetic architecture of personality scores related to handling stress

We find a heritability for handling aggression of approximately 35%. Although heritability is a population-specific property and one thus needs to be careful in drawing comparisons (Lynch and Walsh 1998), this estimate is in line with what has been found in other studies (van Oers and Sinn 2011). The heritability of breath rate (20%) is, however, below this expectation. Nevertheless, the repeatability for both handling aggression and breath rate is equally high, approximately 40% (this paper, cf. Kluen et al. in press). Clearly, the consistency of individuals in terms of handling aggression is largely due to genetic differences between individuals. However, the relatively high repeatability of breath rate is because it is strongly affected by non-heritable, so-called permanent environmental effects, which are of approximately the same strength as the heritable effects. The permanent environment captures factors which are permanently associated to individuals such as maternal effects as well as environmental differences between individuals (e.g., quality of their home ranges). In addition, genetic dominance could contribute to this source of variance. These findings qualitatively mirror the results obtained for these personality traits in blue tit offspring, where breath rate is also less heritable than handling aggression and is more affected by common environmental conditions (Brommer and Kluen 2012).

We found no phenotypic or genetic correlation between handling aggression and breath rate. We expected such a genetic correlation for two reasons. Firstly, these traits are negatively genetically correlated in blue tit nestlings (Brommer and Kluen 2012). Secondly, both traits quantify a behavioral response to the stress of being handled. To some extent, therefore, handling aggression and breath rate can be considered as separate quantifications of the same temperament category “boldness” (sensu Réale et al. 2007), and are therefore expected to be associated. The absence of a genetic correlation, however, demonstrates that different genes are underlying handling aggression and breath rate in adults, and that there is, hence, no constraint on the independent evolution of these traits in adults. This finding is in clear contrast to the general conclusion that, based on a review of literature estimates, multiple behaviors tend to have genetic correlations of such a magnitude that they constrain evolution (Dochtermann and Dingemanse 2013). The observation that the magnitude of the genetic correlation between handling aggression and breath rate changes during ontogeny implies that this genetic correlation is not due to the same pleiotropically acting set of genes. Rather, the genetic correlation is likely caused by linkage disequilibrium, because linkage disequilibium is easier to break up than pleiotropy. The cause of this ontogenetic change in the magnitude of the genetic correlation between breath rate and handling aggression can be related to the development of physiological processes underlying the personality traits, which then cause a “gene-by-age” interaction and may also include the effect of experience as it has an important influence on development (Stamps 2003). In addition, the selective process between the nestling and breeding adult stages may break up this genetic correlation. To date, the question of ontogeny has received little attention in studies on animal personality, despite its pivotal importance in understanding how animal personality is shaped (Stamps and Groothuis 2010a,b2010b).

### Why does handling aggression affect recruitment probability?

The foster father's handling aggression increases the offspring's probability to recruit as a breeding adult in the study area. Offspring recruitment is not associated with the personality of the genetic father, although we have not identified extra-pair offspring and our selection on the genetic father's personality may therefore provide a biased estimate of the true selection. Our measure of fitness is based on the production of local recruits, and is thus sensitive to variation in natal dispersal. However, we find no evidence that an offspring's natal dispersal distance within the study area is associated with its parents' handling aggression. One important component of offspring survival is the predation in the first weeks after fledging (Naef-Daenzer et al. 2001). For males in this population, there is no significant between-individual correlation of handling aggression to the intensity of nest defence of their 16-day-old nestlings (Fresneau et al. in press). Hence, we do not believe that the recruitment benefit of high foster male handling aggression operates via an increased capacity to defend fledged offspring. Rather, offspring fostered by fathers with a high handling aggression obtain a high mass at fledging. In general, a high mass at fledging increases postfledging survival in small passerines (e.g. Garnett 1981; Tinbergen and Boerlijst 1990; Naef-Daenzer et al. 2001; Monrós et al. 2002). Several nonexclusive explanations exist for the positive rearing effect of the foster father's handling aggression on offspring recruitment. Firstly, a male with a high handling aggression score could be a male which is aggressive to conspecifics, which may allow him to obtain a high quality territory. High-quality territories would be beneficial during the rearing of the nestlings, but also during the postfledging period when the offspring stay in their natal territory for some weeks to forage (Nilsson and Smith 1985; Naef-Daenzer et al. 2001). Secondly, males with a high handling aggression score could themselves (independently from territory quality) have a higher feeding rate or provide better quality food items. Blue tit males have an important role in provisioning (Dickens et al. 2008), and tend to have higher provisioning rates than females (Grieco 1999). On the other hand, males with a high handling aggression score could be more attractive for females and their females could invest more effort in taking care of the offspring (Stamps 2003). For example, blue tit males which show high aggression to conspecifics prior to breeding are poor feeders, but are paired with females with a high provisioning rate which leads to high reproductive success for such males (Mutzel et al. 2013). Whether there is a direct or indirect pathway, it is clear that there is scope for variation in the foster fathers' handling aggression to affect the rate his offspring are provisioned and thereby affect nestling development and subsequent recruitment.

Apart from the positive effect of foster male handling aggression on recruitment probability, our study demonstrates that a pair which has mated assortatively in terms of handling aggression also has a better ability to produce recruits. Hence, choosing to mate with a partner with similar handling aggression potentially has clear fitness benefits in blue tits. Blue tits indeed tend to mate assortatively with respect to handling aggression, which could indicate that such mate selection is acting. In general, of course, experimental manipulation is needed to verify that traits which are associated with fitness indeed are the target of selection. In our case, handling aggression is not a trait expressed in the wild, but it may be associated to some aspect of behavior or phenotype of the birds which can be assessed by other individuals in the wild. As pointed out by Schuett et al. (2011), one important caveat to these findings is that rather than individuals selecting each other on the basis of their personality, apparent assortative mating may arise if male and female become more similar to each other after pairing compared to before pairing. By extension, the positive effect of assortative mating of foster parents on recruitment could indicate that individuals, which have managed to “match” each other's handling aggression after pairing, are those which are more successful in rearing their offspring. Because handling aggression is only scored once for the majority of individuals, we cannot, at present, formally exclude this possible interpretation of our results, although it should be noted that handling aggression has a 40% repeatability (35% heritability), which suggests there is not much room for interactions between the members of a pair to alter its expression.

## Conclusion

This study shows that personality traits handling aggression and breath rate are heritable and that natural selection acts on these two traits. Selection acts through adult survival on breath rate in females and through offspring recruitment on male handling aggression. Moreover, selection favors choosing a mate with similar handling aggression score, probably because parents with a matching handling aggression are more capable to rear offspring. Hence, we find that two relatively simple metrics of behavior capture an aspect of personality of relevance to their performance in nature in terms of natural and sexual selection. We do not find temporal fluctuations in the selective forces acting on these aspects of blue tit personality. A further striking finding is that the negative genetic correlation in handling aggression and breath rate found in offspring is not stable over ontogeny as it is absent in adults. Placing the current findings in a more explicit ontogenetic framework where the association of handling aggression and breath rate to other fitness-related traits also is included is needed to elucidate the evolutionary quantitative genetics of these aspects of personality.

## Supporting Information

**Table S1.** Model ranking for capture probability (*p*) assuming full model structure for apparent survival. **Table S2.** Coefficients for the CMR estimated apparent survival (Phi) and capture probability (*p*). **Table S3.** Recruitment probability of nestlings as a function of the breath rate of foster and genetic parents. **Fig. S1.** Plot of the effects of the breath rate of foster and genetic fathers on recruitment probability for the different years included in this study.
