# Supplementary material for: Evolutionary quantitative genetics of behavioral responses to handling in a wild passerine
Source: Ecol Evol. 2014 Jan 20;4(4):427–40. doi: 10.1002/ece3.945 (PMC3936389; doi:10.1002/ece3.945)
Supplement: Supplementary file 1 — Table S1. Model ranking for capture probability (p) assuming full model structure for apparent survival. Table S2. Coefficients for the CMR estimated apparent survival (Phi) and capture probability (p). Table S3. Recruitment probability of nestlings as a function of the breath rate of foster and genetic parents. Fig. S1. Plot of the effects of the breath rate of foster and genetic fathers on recruitment probability for the different years included in this study. [file ece30004-0427-sd1.docx]

**Table S1**. Ranking of the Capture-Mark-Recapture models for capture probability P for handling aggression and breath rate. Models consist of P conditioned on different combinations of the variables handling aggression (agg), year (t) and sex (g) in addition to constant (.). In all cases, the model for apparent survival is the full model (g+t+agg+(g*t)+(g*agg)+ (agg*t)+ (g*t*agg)). ΔAICc is the difference in AIC between the candidate model and the top model.

Handling aggression

| P | parameters | AICc | ΔAICc |
| --- | --- | --- | --- |
| . | 21 | 1602.829 | 0 |
| g | 22 | 1603.8144 | 0.9854 |
| agg | 22 | 1604.2539 | 1.4249 |
| g+agg+(g*agg) | 24 | 1604.5869 | 1.7579 |
| g+agg | 23 | 1604.5963 | 1.7673 |
| t | 25 | 1607.12 | 4.291 |
| g+t+agg+(g*agg) | 28 | 1607.6382 | 4.8092 |
| g+t | 26 | 1608.1045 | 5.2755 |
| agg+t | 26 | 1608.3284 | 5.4994 |
| g+t+agg | 27 | 1608.9221 | 6.0931 |
| g+t+agg+(g*t)+(g*agg) | 32 | 1612.5764 | 9.7474 |
| g+t+(g*t) | 30 | 1613.3288 | 10.4998 |
| g+t+agg+(g*t) | 31 | 1614.0627 | 11.2337 |
| g+t+agg+(g*agg)+ (agg*t) | 32 | 1614.0871 | 11.2581 |
| g+t+agg+(agg*t) | 31 | 1614.7889 | 11.9599 |
| agg+t+(agg*t) | 30 | 1614.933 | 12.104 |
| g+t+agg+(g*t)+(g*agg)+ (agg*t) | 36 | 1620.1895 | 17.3605 |
| g+t+agg+(g*t)+ (agg*t) | 35 | 1620.5098 | 17.6808 |
| g+t+agg+(g*t)+(g*agg)+ (agg*t)+ (g*t*agg) | 40 | 1627.5705 | 24.7415 |

Breath rate

| P | parameters | AICc | D AICc |
| --- | --- | --- | --- |
| . | 21 | 1593.7924 | 0 |
| g | 22 | 1594.6962 | 0.9038 |
| br | 22 | 1595.6338 | 1.8414 |
| g+br | 23 | 1596.5313 | 2.7389 |
| t | 25 | 1597.9724 | 4.18 |
| g+br+(g*br) | 24 | 1598.6028 | 4.8104 |
| g+t | 26 | 1599.0125 | 5.2201 |
| br+t | 26 | 1599.7792 | 5.9868 |
| g+t+br | 27 | 1600.8106 | 7.0182 |
| g+t+br+(g*br) | 28 | 1602.9037 | 9.1113 |
| g+t+(g*t) | 30 | 1604.4186 | 10.6262 |
| g+t+br+(g*t) | 31 | 1606.232 | 12.4396 |
| br+t+(br*t) | 30 | 1607.1376 | 13.3452 |
| g+t+br+(br*t) | 31 | 1608.2084 | 14.416 |
| g+t+br+(g*t)+(g*br) | 32 | 1608.3494 | 14.557 |
| g+t+br+(g*br)+ (br*t) | 32 | 1610.2899 | 16.4975 |
| g+t+br+(g*t)+ (br*t) | 35 | 1613.7149 | 19.9225 |
| g+t+br+(g*t)+(g*br)+ (br*t) | 36 | 1615.6481 | 21.8557 |
| g+t+br+(g*t)+(g*br)+ (br*t)+ (g*t*br) | 40 | 1620.5027 | 26.7103 |

**Table S2**. Coefficients for the CMR estimated apparent survival (Phi) and capture probability (P). The encounter history covered 2007 – 2012, but apparent survival of 2011-2012 cannot be estimated. (a) Coefficients (Beta) and their SE of the effects modeled in the top model for breath rate on the logit scale. The intercept for Phi is the logit of apparent survival in 2007, and the coefficients denoting the change in apparent survival in the other years is denoted by Δ. The coefficient for sex gives the difference of male apparent survival relative to females. Data on breath rate was transformed to zero mean and unit standard deviation, such that the coefficient for ‘br’ gives the slope (on the logit scale) of how apparent survival changes with 1 SD of breath rate. Fig.1 is based on the coeficients reported in panel a. (b) Estimates of apparent survival on the data assuming the individual covariate (handling aggression or breath rate) is equal to its mean. That is, these estimates are for the model Φ(*t* + *s*) P(.).

(a) Parameters on the logit scale.

| Parameter | Beta | Standard Error | Lower 95% CI | Upper 95% CI |
| --- | --- | --- | --- | --- |
| intercept Phi | -0.41 | 0.17 | -0.74 | -0.08 |
| sex (male) | 0.18 | 0.13 | -0.08 | 0.44 |
| Δ 2008 | -0.04 | 0.22 | -0.47 | 0.38 |
| Δ 2009 | 0.76 | 0.22 | 0.33 | 1.18 |
| Δ 2010 | -0.63 | 0.21 | -1.03 | -0.23 |
| Δ 2011 | 0.15 | 0.22 | -0.29 | 0.58 |
| br | -0.27 | 0.09 | -0.44 | -0.10 |
| br*sex (male) | 0.30 | 0.14 | 0.03 | 0.58 |
| intercept P | 1.88 | 0.24 | 1.41 | 2.35 |

(b) Estimates on the data scale

| Parameter | sex | year | Estimate | Standard Error | Lower CI | Upper CI |
| --- | --- | --- | --- | --- | --- | --- |
| Phi 1 | males | 2007 | 0.43 | 0.04 | 0.35 | 0.52 |
| Phi 2 | males | 2008 | 0.63 | 0.04 | 0.54 | 0.71 |
| Phi 3 | males | 2009 | 0.30 | 0.03 | 0.24 | 0.37 |
| Phi 4 | males | 2010 | 0.48 | 0.04 | 0.39 | 0.56 |
| Phi 5 | males | 2011 | 0.44 | 0.04 | 0.36 | 0.52 |
| Phi 6 | females | 2007 | 0.39 | 0.04 | 0.31 | 0.47 |
| Phi 7 | females | 2008 | 0.59 | 0.04 | 0.50 | 0.67 |
| Phi 8 | females | 2009 | 0.26 | 0.03 | 0.21 | 0.32 |
| Phi 9 | females | 2010 | 0.43 | 0.04 | 0.36 | 0.52 |
| Phi 10 | females | 2011 | 0.40 | 0.04 | 0.32 | 0.48 |
| P |  |  | 0.87 | 0.03 | 0.80 | 0.91 |

**Table S3.** Effects of the genetic and foster parents’ breath rates (BR) on offspring recruitment into the breeding population (n=2592 fledglings from 246 broods). The model (GLMM with a binomial error distribution) includes nest of origin and nest of rearing as random effects. All the individual covariates were standardized to zero mean and unit standard deviation. Year is a 4 level factor. P(z) values of estimates are given by a z-test on the coefficients to test whether they differ significantly from 0. Coefficients for Year are in comparison with the year 2007. A Likelihood ratio test (χ²) was used to test the significance of the fixed effects by comparing the Laplace approximated likelihood of models with and without each variable.

The statistically significant two-way interactions “BR Foster Father * Year” and “BR Genetic Father * Year” stem from the strong effects of the breath rate of foster and genetic fathers on recruitment in 2008, but these effect are of opposite direction (plotted in Fig. S2). These effects are difficult to interpret and are here not considered biological meaningful since, under nonexperimental settings, genetic fathers also foster their offspring.

| Effect | Variance | Estimate | SD | ±SE | z | P (z) | χ² | df | P |
| --- | --- | --- | --- | --- | --- | --- | --- | --- | --- |
| Random effects |  |  |  |  |  |  |  |  |  |
| Genetic ID | 0.35 |  | 0.59 |  |  |  | 1.82 | 1.00 | 0.1778 |
| Rear ID | 0.41 |  | 0.64 |  |  |  | 2.75 | 1.00 | 0.10 |
| Fixed effects |  |  |  |  |  |  |  |  |  |
| **Intercept** |  | **-3.00** |  | **0.24** | **-12.65** | **< 0.001** |  |  |  |
| BR Foster father |  | 0.14 |  | 0.31 | 0.46 | 0.65 |  |  |  |
| BR Foster mother |  |  |  |  |  |  | 3.26 | 1 | *0.07* |
| BR Genetic father |  | -0.27 |  | 0.31 | -0.85 | 0.40 |  |  |  |
| BR Genetic mother |  |  |  |  |  |  | 1.07 | 1 | 0.30 |
| BR Foster father * BR Foster mother |  |  |  |  |  |  | 0.69 | 1 | 0.41 |
| BR Genetic father * BR Genetic mother |  |  |  |  |  |  | 1.11 | 1 | 0.29 |
| Year 2008 |  | -0.13 |  | 0.34 | -0.37 | 0.71 |  |  |  |
| **Year 2009** |  | **-1.08** |  | **0.41** | **-2.67** | **0.01** |  |  |  |
| Year 2010 |  | *-0.62* |  | *0.37* | *-1.69* | *0.09* |  |  |  |
| **BR Foster father * Year** |  |  |  |  |  |  | **8.59** | **3** | **0.04** |
| **in 2008** |  | **-1.12** |  | **0.44** | **-2.55** | **0.01** |  |  |  |
| in 2009 |  | 0.07 |  | 0.42 | 0.16 | 0.87 |  |  |  |
| in 2010 |  | -0.25 |  | 0.45 | -0.55 | 0.58 |  |  |  |
| BR Foster mother * Year |  |  |  |  |  |  | 1.34 | 3 | 0.72 |
| **BR Genetic father*Year** |  |  |  |  |  |  | **11.04** | **3** | **0.01** |
| **in 2008** |  | **0.99** |  | **0.39** | **2.54** | **0.01** |  |  |  |
| in 2009 |  | -0.22 |  | 0.44 | -0.50 | 0.62 |  |  |  |
| in 2010 |  | 0.26 |  | 0.45 | 0.59 | 0.55 |  |  |  |
| BR Genetic mother*Year |  |  |  |  |  |  | 2.17 | 3 | 0.54 |
| BR Foster father * BR Foster mother*Year |  |  |  |  |  |  | 3.83 | 3 | 0.28 |
| BR Genetic father * BR Genetic mother*Year |  |  |  |  |  |  | 0.49 | 3 | 0.92 |

**Figure S1**. Plot of the effect of the breath rate of foster and genetic fathers on offspring recruitment for the different study years. The year 2008, which essentially caused the statistically significant interaction between these traits and year (Table S3) is indicated by a red line and the other years in other colours. The effect of breath rate of foster and genetic father on recruitment are opposite in sign and cancel each other out in the usual biological setting where genetic fathers also foster their offspring.
